# Association Between Periconceptional Weight of Maternal Grandmothers and Attention-Deficit/Hyperactivity Disorder in Grandchildren

**DOI:** 10.1001/jamanetworkopen.2021.18824

**Published:** 2021-07-29

**Authors:** Gyeyoon Yim, Andrea Roberts, Alberto Ascherio, David Wypij, Marianthi-Anna Kioumourtzoglou, Marc G. Weisskopf

**Affiliations:** 1 Department of Environmental Health, Harvard T.H. Chan School of Public Health, Boston, Massachusetts; 2 Department of Epidemiology, Harvard T.H. Chan School of Public Health, Boston, Massachusetts; 3 Department of Nutrition, Harvard T.H. Chan School of Public Health, Boston, Massachusetts; 4 Channing Division of Network Medicine, Department of Medicine, Brigham and Women’s Hospital, Boston, Massachusetts; 5 Department of Biostatistics, Harvard T.H. Chan School of Public Health, Boston, Massachusetts; 6 Department of Pediatrics, Harvard Medical School, Boston, Massachusetts; 7 Department of Cardiology, Children’s Hospital Boston, Boston, Massachusetts; 8 Department of Environmental Health Sciences, Mailman School of Public Health, Columbia University, New York, New York

## Abstract

**Importance:**

Neurodevelopmental disorders have been proposed to involve alterations to epigenetic regulation, and epigenetic effects may extend to germline cells to affect later generations. Weight status may affect DNA methylation, and maternal weight before and during pregnancy has been associated with offspring DNA methylation as well as attention-deficit/hyperactivity disorder (ADHD).

**Objective:**

To assess whether a woman’s weight before and during pregnancy is associated with ADHD in her grandchild.

**Design, Setting, and Participants:**

This cohort study analyzed data from 19 835 grandmother-mother dyads and 44 720 grandchildren in the Nurses’ Health Study II (NHS-II) cohort (2001-2013), a population-based prospective cohort study. Cluster-weighted generalized estimating equations were modeled to estimate the association of grandmother’s prepregnancy body mass index (BMI) and gestational weight gain with grandchild risk of ADHD. Data analyses were conducted from May 2018 to April 2021. Grandmothers reported their height and weight before, and weight gain during, their pregnancy with the NHS-II participants. Mothers self-reported height and weight prior to pregnancy. From those data, grandmother BMI and mother BMI were calculated as weight in kilograms divided by height in meters squared and categorized as underweight (<18.5), healthy/normal (18.5-24.9), overweight (25.0-29.9), or obese (≥30).

**Main Outcomes and Measures:**

Cases of ADHD identified by maternal report of having a child with a diagnosis of ADHD.

**Results:**

In total, 19 835 grandmothers (97.6% White race/ethnicity; 2113 [10.7%] prepregnancy underweight and 1391 [7.0%] prepregnancy overweight or obese) were included in this cohort study. Of 44 720 grandchildren, 3593 (8%) received a diagnosis of ADHD. Higher odds of ADHD among grandchildren were found for those whose grandmother was underweight compared with healthy weight prior to pregnancy with the NHS-II participant (adjusted odds ratio, 1.25; 95% CI, 1.10-1.42). By contrast, grandmother gestational weight gain was not significantly associated with risk of grandchild ADHD (adjusted odds ratio for <20 lbs [9.1 kg], 1.06; 95% CI, 0.96-1.16; adjusted odds ratio for >29 lbs [13.2 kg], 1.01; 95% CI, 0.91-1.13). Mother prepregnancy BMI showed an association with ADHD among offspring, with a stronger association detected for obese status (adjusted odds ratio, 1.27; 95% CI, 1.07-1.49) than for overweight status (adjusted odds ratio, 1.13; 95% CI, 1.02-1.26) compared with normal weight as a reference group. The positive association between grandmother prepregnancy underweight and ADHD risk among the grandchildren remained unchanged after further adjustment for potential mediators, including maternal prepregnancy BMI.

**Conclusions and Relevance:**

The results of this cohort study indicate that grandmother underweight prior to pregnancy is associated with an increased risk of ADHD among grandchildren, independent of grandmother gestational weight gain and independent of maternal prepregnancy weight status.

## Key Points

**Question:**

Is there an association of grandmother prepregnancy body mass index or gestational weight gain with risk of attention-deficit/hyperactivity disorder (ADHD) among grandchildren?

**Findings:**

This cohort study of 19 835 grandmother-mother dyads indicated independent, significant associations of grandmother underweight and mother overweight or obesity prior to pregnancy with higher odds of ADHD among 44 720 children in the following generation.

**Meaning:**

The present study findings suggest that underweight grandmaternal periconceptional body mass index may be associated with ADHD among grandchildren, potentially via the germline.

## Teaser

This cohort study analyzes data from mother-daughter dyad participants in the Nurses’ Health Study II to assess whether a woman’s weight before or during pregnancy is associated with attention-deficit/hyperactivity disorder (ADHD) in grandchildren.

## Introduction

Attention-deficit/hyperactivity disorder (ADHD), one of the most common neurodevelopmental disorders,^1^ is characterized by hyperactivity, impulsivity, and inattention.^2,3^ Worldwide estimated ADHD prevalence is approximately 5%.^4,5^ Children with ADHD experience complications such as difficulties in peer relationships, substance abuse, and increased risk for delinquency.^6,7,8^ Identifying risk factors, underlying etiology, and biological factors that may predispose children to ADHD could have great public health importance by informing prevention or treatment efforts.

Although heritability estimates for ADHD are 70% to 80%, the etiology of ADHD still appears partially attributable to nongenetic factors.^9,10,11^ Maternal prepregnancy obesity and excessive gestational weight gain (GWG) have been associated with increased risk of ADHD or symptoms related to ADHD in offspring.^12,13,14,15,16,17,18,19,20^ However, there is increasing interest in the possibility of multigenerational effects of pregnancy exposures. Peripregnancy maternal weight can affect endocrine function^21^ and DNA methylation,^22^ both of which can affect the germline and are implicated in multigenerational effects of exposures.^23^ Animal studies have found evidence for such germline effects of weight-related variables around pregnancy,^24,25,26^ with possible implications for ADHD.^27,28^ Human studies have also found that maternal prepregnancy body mass index (BMI) and GWG may be associated with epigenetic effects in offspring.^29,30^

Studies of grandmothers’ exposures and associated outcomes in grandchildren are inherently challenging, mainly owing to data collection over multiple generations.^31^ The objective of the present study was to assess the possible multigenerational associations of prepregnancy BMI and GWG with ADHD by using the Nurses’ Health Study II (NHS-II), a large, well-characterized longitudinal study of nurses.

## Methods

### Study Population

The NHS-II is a prospective cohort study of 116 430 US registered female nurses who were born between 1946 and 1964.^32^ In 2001, 39 904 nurses’ mothers who were alive and free of cancer in 2000 were enrolled in the Nurses’ Mothers’ Cohort Study (NMCS) and provided additional information on the prenatal and childhood environment of the nurses.^33^ In the present study, we refer to the nurses as generation 1 (G1), their mothers as generation 0 (G0), and their children as generation 2 (G2). This study followed the Strengthening the Reporting of Observational Studies in Epidemiology (STROBE) reporting guideline for cohort studies. Our study was approved by the institutional review board of Brigham and Women’s Hospital and Harvard T.H. Chan School of Public Health, Boston, Massachusetts. Returning completed questionnaire is considered evidence of informed consent in a manner consistent with the Common Rule requirements at enrollment in 1989, and participants have since completed biennial questionnaires. No one received compensation or was offered any incentive for participating in this study.

Our analytic population was first restricted to 23 898 G1 nurses who had children, returned the 2013 NHS-II questionnaire, were not themselves adopted, and whose mother participated in the NMCS. We then additionally excluded 1814 G1 nurses for whom data for G0 prepregnancy BMI or GWG were missing. We excluded G2 children with a sibling born in the same year to the same mother because if the mother reported a child with ADHD, we would not know which child it was. The final analytic sample included 19 835 G0-G1 dyads and 44 720 G2 offspring. Although the NMCS is a subset of the NHS-II, we found little difference in G2 ADHD prevalence by G0 NMCS membership status (7.9% of NMCS participants vs 7.2% of NMCS nonparticipants), suggesting that selection bias was unlikely.

### Exposure Assessment

In 2001, the NMCS G0 participants reported their height and weight prior to the pregnancy with the NHS-II nurse (G1). From those data, we calculated BMI (calculated as weight in kilograms divided by height in meters squared), which we categorized as underweight (<18.5), healthy/normal (18.5-24.9), overweight (25.0-29.9), and obese (≥30).^34^ The G0 grandmothers also reported weight gain during the pregnancy with the G1 nurse, with categorical responses for GWG of less than 10 lb (<4.5 kg), 10 to 14 lb (4.5-6.4 kg), 15 to 19 lb (6.8-8.6 kg), 20 to 29 lb (9.1-13.2 kg), 30 to 40 lb (13.6-18.1 kg), more than 40 lb (>18.1 kg), or “don’t remember.”^33,35^ In our study, the majority (40%) of G0 grandmothers reported weight gain of 20 to 29 lb (9.1-13.2 kg) during pregnancy, which is consistent with the reported mean GWG of that era.^36,37,38,39,40^ We categorized G0 GWG as less than 20 lb (<9.1 kg), 20-29 lb (9.1-13.2 kg), more than 29 lb (>13.2 kg), or no response, which roughly reflected less than, equal to, and more than the recommended GWG in that era.^36,37,38,39,40^ We also considered the nurse’s self-reported BMI prior to the pregnancy with her G2 child as an exposure, with the same prepregnancy BMI categories as for G0.

### Outcome Assessment

In 2013, the G1 nurses were asked “have any of your biological children been diagnosed with attention-deficit/hyperactivity disorder (ADHD)?” and if so, the year of birth of the child(ren). Our main analyses considered identified ADHD cases. The nurses were also asked about children with ADHD on the 2005 questionnaire, but not their year of birth; thus, the specific child with ADHD could not always be identified. In sensitivity analyses, we excluded any ADHD cases identified in 2013 by mothers who did not report a child with ADHD in 2005. Details of the validation study of ADHD diagnosis have been described.^41,42^

### Covariates

Potential confounders were identified by drawing directed acyclic graphs based on previous literature. We considered G0 grandmother self-reported race/ethnicity (White or non-White, because more than 97% of the grandmothers were White), grandmother educational level (high school or less vs college or more), smoking and alcohol use (separately) during pregnancy with the nurse (yes or no), and the grandfather’s educational level (same categories) and occupation (blue collar [eg, sales or clerical, service, craft worker, machine operator or assembler, or military], laborer, farmer, professional [eg, executive manager, administrator, teacher, librarian, doctor, lawyer, or nurse], or did not work). To account for increasing secular trends in obesity and ADHD prevalence,^43,44^ we included G1 birth year. We also considered G1 reporting of her mother’s (G0) lifetime history of major depression (yes or no) given its association with both overweight or obesity and ADHD.^45,46,47,48^ In additional analyses, we also considered G1 pregnancy-related complications (ie, preeclampsia or toxemia, pregnancy-related high blood pressure, or gestational diabetes; yes or no) while pregnant with the G2 child^49,50^ and her child’s birth weight (<5.5 lb [<2.5 kg], 5.5-9.9 lb [2.5-4.5 kg], or ≥10 lb [>4.5 kg])^51^ as potential mediator variables.

### Statistical Analysis

Overweight or obesity may disrupt the reproductive functions of a woman, potentially affecting the number of G2 children for a given G1 nurse in the present study,^52,53^ which was associated with ADHD in our data (eTable 1 in the Supplement), leading to possible informative clustering.^54,55^ Thus, we used cluster-weighted generalized estimating equations with a logit link^55^ to estimate odds ratios (ORs) and 95% CIs for G2 ADHD by G0 pregnancy-related weight characteristics, adjusting for covariates. Those equations weight by the inverse of the number of children for each nurse (therefore, grandmother too) to handle informative clustering. Generalized estimation equations simultaneously account for potential unknown correlations between outcomes among grandchildren born to the same nurse. A compound symmetric working covariance structure was applied, assuming constant correlation regardless of the order of children for each nurse. We adjusted for the described nonmediator covariates.

We conducted the following sensitivity analyses. First, we investigated whether the distribution of key G1-reported covariates differed according to the G0 NMCS membership status. Second, we excluded cases in which G1 mothers did not report a child with ADHD in 2005. Third, we considered differences by child sex by stratifying on G2 sex. Fourth, we examined how much of a potential association of our exposure with ADHD was accounted for by the described mediators by including those terms in the model. In addition, by having G0 prepregnancy BMI and GWG and G1 prepregnancy BMI in a model together, we were able to assess the extent to which G1 prepregnancy BMI mediated any association between G0 prepregnancy BMI or GWG and G2 ADHD, and the extent to which G0 GWG mediated any association with G0 prepregnancy BMI. Such checks on mediation are valid only if there is no interaction between the exposure of interest and the mediator,^56^ which we verified by running models that included these interaction terms and evaluating their significance. Fifth, we carried out a quantitative bias analysis to assess the influence of possible exposure misclassification.^57^ Complete case analysis was conducted given the small amount of missing data (generally <5%). All analyses were performed from May 2018 to April 2021 using SAS (SAS Institute Inc) or the R package episensr, version 4.0.4 (R Development Core Team), for the quantitative bias analysis. A 2-sided value of *P* ≤ .05 was considered statistically significant.

## Results

Of 19 835 G0 grandmothers, before pregnancy, 2113 (10.7%) had underweight and 1391 (7.0%) had overweight or obesity (Table 1). During pregnancy, 6572 (33.1%) G0 grandmothers gained less than 20 lb (9.1 kg), and 4276 (21.6%) gained more than 29 lb (13.2 kg) (Table 2). Prepregnancy BMI was higher for G1 (11.7% with overweight; 3.7% with obesity; and only 1.8% with underweight) than for G0. In general, G0 grandmothers either with underweight or with overweight or obesity had characteristics that tended to reflect lower socioeconomic status (Table 1). Grandmothers with GWG more than 29 lb (13.2 kg) also more frequently had a lifetime history of depression than those with GWG of 29 lb or less (Table 2). Approximately 8.0% (n = 3593) of G2 grandchildren in the study sample had ADHD.

**Table 1.** Characteristics of G0 (N = 19 835), G1 (N = 19 835), and G2 (N = 44 720) by G0 Prepregnancy BMI

Characteristic	G0 Prepregnancy BMI, No. (%)		
	<18.5 (n = 2113)	18.5-24.9 (n = 16 331)	≥25.0 (n = 1391)
**G0 generation**			
Race/ethnicity			
White	2062 (97.6)	16 041 (98.2)	1360 (97.8)
Non-White	51 (2.4)	290 (1.8)	31 (2.2)
Grandmother educational level			
High school or less	1428 (67.6)	9994 (61.2)	940 (67.6)
College or more	685 (32.4)	6337 (38.8)	451 (32.4)
Grandfather educational level			
High school or less	1188 (56.2)	8990 (55.1)	1003 (72.1)
College or more	925 (43.8)	7341 (45.0)	388 (27.9)
Grandfather occupation			
Blue collar^a^	1225 (58.0)	8962 (54.9)	823 (59.2)
Laborer	148 (7.0)	913 (5.6)	145 (10.4)
Farmer	87 (4.1)	1161 (7.1)	177 (12.7)
Professional^b^	640 (30.3)	5233 (32.0)	243 (17.5)
None	13 (0.6)	62 (0.4)	3 (0.2)
Grandmother smoking during pregnancy with G1	665 (31.5)	4260 (26.1)	252 (18.1)
Grandmother alcohol use during pregnancy with G1	620 (29.3)	5517 (33.8)	340 (24.4)
Grandmother weight gain during pregnancy with G1			
<20 lb (<9.1 kg)	735 (34.8)	5339 (32.7)	498 (35.8)
20-29 lb (9.1-13.2 kg)	735 (34.8)	6737 (41.3)	514 (37.0)
>29 lb (>13.2 kg)	550 (26.0)	3418 (20.9)	308 (22.1)
Don’t remember	93 (4.4)	837 (5.1)	71 (5.1)
Grandmother lifetime history of depression^c^	201 (9.5)	1427 (8.7)	117 (8.4)
Grandmother birth year, mean (SD)	1930.2 (6.4)	1928.9 (6.4)	1928.4 (6.6)
Grandmother age at G1 birth, mean (SD), y	24.6 (4.5)	26.2 (4.9)	27.2 (5.1)
**G1 generation**			
Birth weight			
<5.5 lb (<2.5 kg)	186 (8.8)	914 (5.6)	55 (4.0)
5.5-9.9 lb (2.5-4.5 kg)	1751 (82.9)	13 876 (85.0)	1176 (84.5)
≥10 lb (>4.5 kg)	3 (0.1)	131 (0.8)	38 (2.7)
Missing	173 (8.2)	1410 (8.6)	122 (8.8)
Birth year, mean (SD)	1954.8 (4.7)	1955.1 (4.6)	1955.6 (4.6)
**G2 generation**			
Total No.	4712	36 915	3093
No. of G2 offspring per G1 mother, median (range)^d^	2 (1-8)	2 (1-12)	2 (1-7)
Sex			
Girls	2155 (45.7)	17 065 (46.2)	1404 (45.4)
Boys	2370 (50.3)	18 237 (49.4)	1530 (49.5)
Missing	187 (4.0)	1613 (4.4)	159 (5.1)
Prenatal exposure to G1 prepregnancy BMI			
<18.5	195 (4.1)	479 (1.3)	7 (0.2)
18.5-24.9	3908 (82.9)	29 391 (79.6)	2033 (65.7)
25.0-29.9	492 (10.4)	5306 (14.4)	685 (22.2)
≥30	117 (2.5)	1739 (4.7)	368 (11.9)
Birth weight			
<5.5 lb (<2.5 kg)	194 (4.1)	1124 (3.0)	105 (3.4)
5.5-9.9 lb (2.5-4.5 kg)	4385 (93.1)	34 552 (93.6)	2866 (92.7)
≥10 lb (>4.5 kg)	105 (2.2)	968 (2.6)	98 (3.2)
Missing	28 (0.6)	271 (0.7)	24 (0.8)
G1 pregnancy complications^e^	404 (8.6)	3030 (8.2)	302 (9.8)
G1 age at G2 birth, mean (SD), y	29.0 (5.0)	29.4 (4.9)	29.2 (5.1)
Birth year, mean (SD)	1983.9 (7.5)	1984.6 (7.3)	1984.9 (7.4)

Abbreviations: BMI, body mass index (calculated as weight in kilograms divided by height in meters squared); G0, generation 0 (grandmothers); G1, generation 1 (nurses); G2, generation 2 (grandchildren).

^a^ Sales, clerical, secretary, service worker, craft worker, machine operator or assembler, or military.

^b^ Executive manager, administrator, teacher, or librarian.

^c^ Reported by G1 daughters.

^d^ As of 2009.

^e^ Preeclampsia or toxemia, pregnancy-related high blood pressure, or gestational diabetes.

**Table 2.** Characteristics of G0 (N = 19 835), G1 (N = 19 835), and G2 (N = 44 720) by G0 GWG

Characteristic	G0 GWG, No. (%)			
	<20 lb (<9.1 kg) (n = 6572)	20-29 lb (9.1-13.2 kg) (n = 7986)	>29 lb (>13.2 kg) (n = 4276)	Don’t remember (n = 1001)
**G0 generation**				
Race/ethnicity				
White	6438 (98.0)	7866 (98.5)	4208 (98.4)	951 (95.0)
Non-White	134 (2.0)	120 (1.5)	68 (1.6)	50 (5.0)
Grandmother educational level				
High school or less	3874 (59.0)	4937 (61.8)	2870 (67.1)	681 (68.0)
College or more	2698 (41.1)	3049 (38.2)	1406 (32.9)	320 (32.0)
Grandfather educational level				
High school or less	3604 (54.8)	4389 (55.0)	2582 (60.4)	606 (60.5)
College or more	2968 (45.2)	3597 (45.0)	1694 (39.6)	395 (39.5)
Grandfather occupation				
Blue collar^a^	3614 (55.0)	4323 (54.1)	2555 (59.8)	518 (51.8)
Laborer	361 (5.5)	465 (5.8)	308 (7.2)	72 (7.2)
Farmer	519 (7.9)	518 (6.5)	264 (6.2)	124 (12.4)
Professional^b^	2049 (31.2)	2647 (33.2)	1135 (26.5)	285 (28.5)
None	29 (0.4)	33 (0.4)	14 (0.3)	2 (0.2)
Grandmother smoking during pregnancy with G1	1712 (26.1)	2076 (26.0)	1200 (28.1)	189 (18.9)
Grandmother alcohol use during pregnancy with G1	2057 (31.3)	2780 (34.8)	1366 (32.0)	274 (27.4)
Grandmother lifetime history of depression^c^	568 (8.6)	675 (8.5)	410 (9.6)	92 (9.2)
Grandmother birth year, mean (SD)	1928.4 (6.4)	1929.3 (6.3)	1930.1 (6.3)	1925.6 (6.3)
Grandmother age at G1 birth, mean (SD), y	26.5 (5.0)	26.1 (4.7)	25.0 (4.7)	27.8 (5.4)
**G1 generation**				
Birth weight				
<5.5 lb (<2.5 kg)	604 (9.2)	324 (4.1)	154 (3.6)	73 (7.3)
5.5-9.9 lb (2.5-4.5 kg)	5358 (81.5)	6943 (86.9)	3690 (86.3)	812 (81.1)
≥10 lb (>4.5 kg)	38 (0.6)	55 (0.7)	65 (1.5)	14 (1.4)
Missing	572 (8.7)	664 (8.3)	367 (8.6)	102 (10.2)
Birth year, mean (SD)	1955.0 (4.6)	1955.4 (4.6)	1955.1 (4.7)	1953.4 (4.6)
**G2 Generation**				
Total No.	14 765	18 146	9592	2217
No. of G2 offspring per G1 mother, median (range)^d^	2 (1-12)	2 (1-10)	2 (1-8)	2 (1-7)
Sex				
Girls	6782 (45.9)	8419 (46.4)	4429 (46.2)	994 (44.8)
Boys	7341 (49.7)	8903 (49.1)	4753 (49.6)	1140 (51.4)
Missing	642 (4.4)	824 (4.5)	410 (4.3)	83 (3.7)
Prenatal exposure to G1 prepregnancy BMI				
<18.5	245 (1.7)	298 (1.6)	109 (1.1)	29 (1.3)
18.5-24.9	11 674 (79.1)	14 391 (79.3)	7429 (77.5)	1838 (82.9)
25.0-29.9	2123 (14.4)	2559 (14.1)	1521 (15.9)	280 (12.6)
≥30	723 (4.9)	898 (5.0)	533 (5.6)	70 (3.2)
Birth weight				
<5.5 lb (<2.5 kg)	531 (3.6)	533 (2.9)	288 (3.0)	71 (3.2)
5.5-9.9 lb (2.5-4.5 kg)	13 779 (93.3)	17 017 (93.8)	8928 (93.1)	2079 (93.8)
≥10 lb (>4.5 kg)	351 (2.4)	470 (2.6)	301 (3.1)	49 (2.2)
Missing	104 (0.7)	126 (0.7)	75 (0.8)	18 (0.8)
G1 pregnancy complications^e^	1265 (8.5)	1459 (8.0)	842 (8.8)	170 (7.7)
G1 age at G2 birth, mean (SD), y	29.4 (5.0)	29.4 (4.9)	29.2 (5.0)	29.0 (4.9)
Birth year, mean (SD)	1984.5 (7.3)	1984.9 (7.3)	1984.4 (7.4)	1982.6 (7.4)

Abbreviations: BMI, body mass index (calculated as weight in kilograms divided by height in meters squared; G0, generation 0 (grandmothers); G1, generation 1 (nurses); G2, generation 2 (grandchildren); GWG, gestational weight gain.

^a^ Sales, clerical, secretary, service worker, craft worker, machine operator or assembler, or military.

^b^ Executive manager, administrator, teacher, or librarian.

^c^ Reported by G1 daughters.

^d^ As of 2009.

^e^ Preeclampsia or toxemia, pregnancy-related high blood pressure, or gestational diabetes.

We found grandmaternal underweight to be associated with increased odds of ADHD in G2 (adjusted OR, 1.25; 95% CI, 1.10-1.42), but no association with overweight (adjusted OR, 0.99; 95% CI, 0.84-1.17) nor with GWG (adjusted OR for <20 lbs [9.1 kg], 1.06; 95% CI, 0.96, 1.17; adjusted OR for >29 lbs [13.2 kg], 1.02; 95% CI, 0.91-1.14) (Table 3). (There was little difference compared with unadjusted models; eTable 2 in the Supplement.) When both G0 prepregnancy BMI and GWG were included in the same model, the results remained materially unchanged. By contrast, there was a monotonically increasing association between greater maternal (G1) prepregnancy BMI and increased odds of G2 ADHD (adjusted OR for BMI 25.0-29.9, 1.13; 95% CI, 1.02-1.26; and adjusted OR for BMI ≥30, 1.27; 95% CI, 1.07-1.49) (Table 4). When we considered all the G0 and G1 weight variables in the same model, the results were essentially unchanged for the direct association of G0 prepregnancy BMI and G0 GWG with G2 ADHD and for the total association of G1 prepregnancy BMI with G2 ADHD (Figure). Unadjusted results were similar (eTable 3 in the Supplement).

**Table 3.** Odds for ADHD in G2 by G0 Joint Exposure to Prepregnancy BMI and GWG, for 19 835 G1 Mothers in the Nurses’ Mothers’ Cohort Study

Exposure	G2, No.	ADHD cases, No. (%)	Model 1^a^^,^^b^		Model 2^b^^,^^c^		Model 3^b^^,^^d^	
			OR (95% CI)	*P* value	OR (95% CI)	*P* value	OR (95% CI)	*P* value
G0 prepregnancy BMI								
<18.5	4712	442 (9.4)	1.25 (1.10-1.42)	.001	NA	NA	1.25 (1.10-1.42)	<.001
18.5-24.9	36 915	2903 (7.9)	1 [Reference]		NA	NA	1 [Reference]	
≥25.0	3093	248 (8.0)	0.99 (0.84-1.17)	.90	NA	NA	0.99 (0.84-1.16)	.88
G0 GWG								
<20 lb (<9.1 kg)	14 765	1211 (8.2)	NA	NA	1.06 (0.96-1.17)	.23	1.06 (0.96-1.16)	.27
20-29 lb (9.1-13.2 kg)	18 146	1436 (7.9)	NA	NA	1 [Reference]		1 [Reference]	
>29 lb (>13.2 kg)	9592	780 (8.1)	NA	NA	1.02 (0.91-1.14)	.74	1.01 (0.91-1.13)	.85
Don’t remember	2217	166 (7.5)	NA	NA	1.13 (0.92-1.38)	.24	1.13 (0.92-1.39)	.24

Abbreviations: ADHD, attention-deficit/hyperactivity disorder; BMI, body mass index (calculated as weight in kilograms divided by height in meters squared); G0, generation 0 (grandmothers); G1, generation 1 (nurses); G2, generation 2 (grandchildren); GWG, gestational weight gain; NA, not applicable; OR, odds ratio.

^a^ Model 1 presents the total association of G0 prepregnancy BMI with G2 ADHD.

^b^ All models are adjusted for grandmother (G0) race/ethnicity, grandmother and grandfather educational levels, grandfather occupation, grandmother smoking and alcohol use during pregnancy and lifetime history of depression, and maternal (G1) year of birth.

^c^ Model 2 presents the total association of G0 GWG with G2 ADHD when confounding by G0 prepregnancy BMI was not accounted for.

^d^ Model 3 presents the direct association of G0 prepregnancy BMI associated with G2 ADHD accounting for G0 GWG and the total association of G0 GWG with G2 ADHD.

**Table 4.** Odds for ADHD in G2 by G1 Prepregnancy BMI, for the 19 835 G1 Mothers in the Nurses’ Mothers’ Cohort Study

G1 prepregnancy BMI	G2, No.	ADHD cases, No. (%)	Odds ratio (95% CI)			
			Unadjusted	*P* value	Adjusted^a^	*P* value
<18.5	681	49 (7.2)	0.77 (0.56-1.06)	.10	0.72 (0.53-0.99)	.05
18.5-24.9	35 332	2693 (7.6)	1 [Reference]		1 [Reference]	
25.0-29.9	6483	610 (9.4)	1.19 (1.08-1.32)	<.001	1.13 (1.02-1.26)	.02
≥30.0	2224	241 (10.8)	1.38 (1.18-1.63)	<.001	1.27 (1.07-1.49)	.005

Abbreviations: ADHD, attention-deficit/hyperactivity disorder; BMI, body mass index (calculated as weight in kilograms divided by height in meters squared); G1, generation 1 (mothers); G2, generation 2 (grandchildren).

^a^ Adjusted for grandmother (G0) race/ethnicity, grandmother and grandfather educational levels, grandfather occupation, grandmother lifetime history of depression, maternal (G1) year of birth, and G1 smoking status at baseline.

**Figure.**
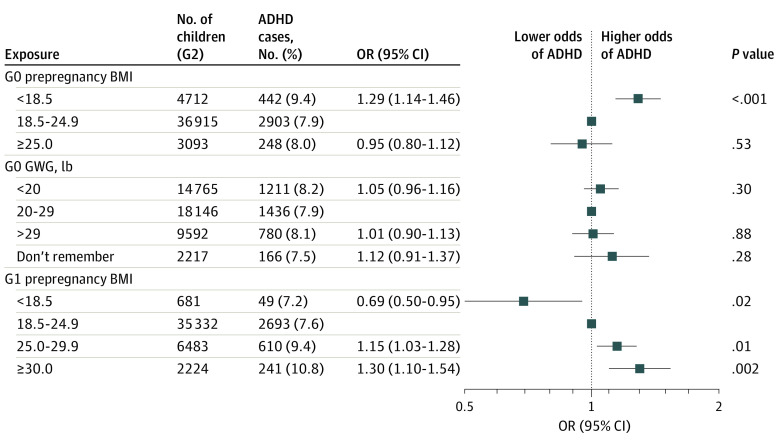
Joint Associations of Grandmother and Mother Weights Before and During Pregnancy With Child Attention-Deficit/Hyperactivity Disorder (ADHD) Estimated odds ratios (ORs) and 95% CIs for ADHD in the third generation in the Nurses’ Mothers’ Cohort Study by the joint exposure of grandmothers (G0) to prepregnancy body mass index (BMI; calculated as weight in kilograms divided by height in meters squared) and gestational weight gain (GWG), controlling for maternal (G1) prepregnancy BMI. Adjusted for grandmother race/ethnicity, grandmother and grandfather educational levels, grandfather occupation, grandmother smoking and alcohol use during pregnancy, grandmother lifetime history of depression, and maternal (G1) year of birth.

In sensitivity analyses, no evidence of selection bias was found owing to loss to follow-up in our data, given the similar distribution of key G1-reported variables according to the NMCS membership. The results were essentially unchanged when we excluded cases in which mothers did not report a child with ADHD in 2005. Although there was little difference in results by G2 sex for the G0 exposures, G1 prepregnancy overweight or obesity was more strongly associated with risk of ADHD among girls than boys (eTable 4 in the Supplement). In the examination of the joint association of G0 prepregnancy BMI, G0 GWG, and G1 prepregnancy BMI with G2 ADHD, results were similar with further adjustment for G1 pregnancy-related complications with G2 and G2 birth weight as potential mediators, except that the association between G1 prepregnancy obesity and risk of ADHD in G2 was slightly attenuated (eTable 5 in the Supplement). Given the null findings for G0 GWG, only G0 prepregnancy BMI was considered in the quantitative bias analysis (eTables 6 and 7 in the Supplement). We found that the misclassification bias-corrected OR for a binary G0 prepregnancy BMI underweight variable could be as high as 5.35 (95% CI, 2.34-12.27, estimated by bootstrapping) with sensitivity of 0.50 and specificity of 0.90, compared with the observed OR of 1.20 (95% CI, 1.08-1.33).

## Discussion

In this large, prospective cohort study, we found that a G0 grandmother being underweight prior to pregnancy with a G1 nurse was associated with increased risk of ADHD in G2 offspring compared with G0 entering pregnancy at healthy weight. The G0 GWG was not associated with ADHD in G2. In contrast to the G0 findings, higher G1 prepregnancy BMI had a monotonically increasing association with higher risk of ADHD in G2. In the model that simultaneously adjusted for G0 prepregnancy BMI, G0 GWG, and G1 prepregnancy BMI, the associations remained identical with each factor modeled separately, suggesting that G0 and G1 weights before pregnancy were associated with risk of G2 ADHD via different pathways, and that the association with G0 prepregnancy underweight was not mediated through G0 GWG. Furthermore, the association with G0 prepregnancy BMI was not substantially changed after adjusting for potential mediating factors (G1 pregnancy-related complications during pregnancy with G2 and G2 birth weight). By contrast, the association between G1 prepregnancy obesity and G2 risk of ADHD was slightly attenuated, suggesting that this association could partially operate via these potential mediators.

Previous research has found evidence for maternal prepregnancy obesity as a risk factor for ADHD, with a recent meta-analysis^58^ indicating that mothers with overweight (adjusted hazard ratio, 1.21; 95% CI, 1.19-2.25) or obesity (adjusted hazard ratio, 1.60; 95% CI, 1.55-1.65) prior to pregnancy were at increased risk for ADHD. Potential underlying biological mechanisms include oxidative stress and inflammation or dysregulation of hormone signaling in the developing brain by maternal obesity prior to pregnancy.^59^ By contrast, there is a paucity of literature about maternal prepregnancy underweight in relation to ADHD in the offspring. A Danish National Birth Cohort study^13^ reported increased risk of autism spectrum disorder (hazard ratio, 1.30; 95% CI, 1.01-1.69), but not of ADHD, among mothers who were underweight prior to pregnancy. Deardorff et al^60^ found prepregnancy underweight to be associated with higher total Behavior Problems Index and externalizing scores only among boys. Studies examining the association of GWG with the child’s (G2) risk of ADHD have shown limited and conflicting findings.^14,18,61^

Several mechanisms may underlie the observed association between G0 prepregnancy underweight and G2 ADHD. Grandmothers who were underweight before pregnancy were more likely to have lower socioeconomic status, other factors of which could be associated with G2 ADHD. However, we adjusted for many G0-level socioeconomic status variables; furthermore, gaining more weight than recommended during pregnancy was also associated with lower socioeconomic status, yet no association was observed for that group. Similarly, grandmaternal prepregnancy underweight may be associated with G1 or G2 pregnancy factors, such as offspring weight and size,^62,63,64^ which may also be associated with ADHD.^65,66^ However, the association with G0 prepregnancy weight status remained robust to further adjustment for G2 birth weight and other G1 and G2 pregnancy factors, which suggests that these factors are not responsible for the association with G0 prepregnancy weight. Increased risk of ADHD in G2 from G0 with prepregnancy underweight may plausibly occur through G1 assortative mating (whereby G1 with more ADHD symptoms choose partners with more ADHD symptoms, possibly increasing the genetic predisposition for ADHD in G2 offspring, as evidence has shown for autism^67,68^) if G0 prepregnancy underweight leads to increased ADHD symptoms in G1. However, this explanation seems unlikely because we found lower—not higher—odds of ADHD in G2 for G1 with prepregnancy underweight (ie, the opposite association). Unfortunately, no data were available regarding ADHD diagnosis or symptoms in G1 nurses.

Alternatively, a plausible underlying biological mechanism involves direct exposure of G2 germ cells to the G0 pregnancy milieu and epigenetic modifications.^69^ In humans, the precursors of eggs and sperm are primordial germ cells (PGCs), the fate of which is induced on embryo implantation in the uterine wall. During early embryogenesis, PGCs are formed and actively migrate to the gonadal ridge. This phase is followed by a second phase in which PGCs initiate controlled cell division directed by environmental cues.^70^ The PGCs lack protection from epigenetic dysregulation by environmental toxicants and thus remain vulnerable to damage during early development.^71^ When a grandmother (G0) experiences unhealthy weight around her pregnancy, both the G1 embryo and G2 germ cell precursors are directly exposed to the G0 pregnancy milieu and signals related to G0 weight status.^72^ Grandmother prepregnancy underweight could influence the epigenetics of the G2 germ cells, such as DNA methylation patterns,^73^ leading to neurodevelopmental deficits in that generation.

### Limitations and Strengths

This study has limitations. First, outcome misclassification is possible. The ADHD case ascertainment was based on nurses’ reports, rather than on medical records. However, maternal reports of ADHD diagnoses in their children have been found to be reliable,^74^ which was also suggested in our validation study.^41^ Second, exposure misclassification is also likely. The G0 prepregnancy weight, height, and total GWG data were self-reported. In a separate NHS validation study,^75^ maternal prepregnancy weight (*r* = 0.86) and height (*r* = 0.90) were found to be accurate compared with external data collected during their pregnancies, whereas recall of weight gained during pregnancy was only modestly correlated (*r* = 0.42). However, it is unlikely that grandmaternal BMI and GWG reporting differed according to grandchild ADHD diagnosis given that the data were collected from G0 many years before asking the nurses (G1) about ADHD in their children (G2) and there was not yet public awareness about potential multigenerational associations of grandmother weight around pregnancy with neurodevelopmental disorders in grandchildren. Third, obesity and ADHD may share common genetic variants.^76^ However, different patterns of association were found between G0 and G1 prepregnancy BMI in relation to G2 ADHD, which would have been similar if our findings were largely attributable to common genetic confounding between obesity and ADHD. Furthermore, the association between G0 prepregnancy BMI and ADHD risk in G2 was robust after adjusting for G1 BMI prior to pregnancy with G2, presumably partially blocking potentially unmeasured confounding factors due to common genetic susceptibility. Fourth, although our mediation analysis suggested a direct association between G0 prepregnancy BMI and G2 ADHD, our findings should be interpreted with caution given possible measurement error of the mediators.^77^ Fifth, as in all observational studies, the possibility of residual confounding remains, although we were able to adjust for a number of variables.

This study has several strengths. The large, prospective cohort of nurses provided a unique setting to investigate multigenerational associations by including information from 3 generations. A variety of covariates that were directly obtained from G0 gives us more confidence to address potential confounding factors compared with previous studies evaluating multigenerational associations, and data on the pregnancies with G2 enabled us to explore possible mediators of the G0 association.

## Conclusions

This cohort study provides novel evidence showing an association between grandmother weight status around pregnancy and increased risk of ADHD in their grandchildren. This study contributes important information to a growing body of literature on multigenerational associations in humans, suggesting that maternal weight around conception may be associated with neurodevelopment of the third generation. Our findings suggest a different pattern of associations with grandmother peripregnancy weight characteristics than with mother peripregnancy weight characteristics. Animal studies may help elucidate potential underlying biological pathways. Given emerging evidence of multigenerational associations with in utero exposure to various factors,^78,79,80,81,82,83,84,85,86,87,88,89,90,91^ future research should assess risks to germline cells following different exposures.

## Notes

Supplement. **eTable 1.** Prevalence of ADHD by Number of Children (Cluster Size) in the Nurses’ Health Study II, Among the 19,835 G1 **eTable 2.** Unadjusted Odds Ratios (ORs) and 95% Confidence Intervals (CIs) for ADHD in the Third Generation (G2) by Grandmother’s (G0) Joint Exposure to Pre-Pregnancy BMI and GWG, Among the 19,835 Mothers (G1) in the Nurses’ Mothers’ Cohort Study **eTable 3.** Unadjusted Odds Ratios (ORs) and 95% Confidence Intervals (CIs) for ADHD in the Third Generation (G2) by Grandmother’s (G0) Joint Exposure to Pre-Pregnancy BMI and GWG, Adjusting for Maternal (G1) Pre-Pregnancy BMI, Among the 19,835 Mothers (G1) in the Nurses’ Mothers’ Cohort Study **eTable 4.** Odds Ratios (ORs) and 95% Confidence Intervals (CIs) for ADHD in the Third Generation (G2) by Grandmother’s (G0) Joint Exposure to Pre-Pregnancy BMI and GWG, Adjusting for Maternal (G1) Pre-Pregnancy BMI, Among the 19,835 Mothers (G1) in the Nurses’ Mothers’ Cohort Study, Stratified by G2 Sex **eTable 5.** Odds Ratios (ORs) and 95% Confidence Intervals (CIs) for ADHD in the Third Generation (G2) by Grandmother’s (G0) Joint Exposure to Pre-Pregnancy BMI and GWG, Adjusting for Maternal (G1) Pre-Pregnancy BMI and Other Potential Mediators, Among the 19,835 Mothers (G1) in the Nurses’ Mothers’ Cohort Study **eTable 6.** Data for the Quantitative Bias Analysis of Grandmother Pre-Pregnancy Weight and Grandchild ADHD Status **eTable 7.** Quantitative Bias Analysis to Assess the Impact of Nondifferential Misclassification of Grandmother (G0) Pre-Pregnancy Underweight in Relation to Grandchild (G2) ADHD in the Nurses’ Mothers’ Cohort Study
