# Supplementary material for: Association Between Periconceptional Weight of Maternal Grandmothers and Attention-Deficit/Hyperactivity Disorder in Grandchildren
Source: JAMA Netw Open. 2021 Jul 29;4(7):e2118824. doi: 10.1001/jamanetworkopen.2021.18824 (PMC8322994; doi:10.1001/jamanetworkopen.2021.18824)
Supplement: Supplement. — eTable 1. Prevalence of ADHD by Number of Children (Cluster Size) in the Nurses’ Health Study II, Among the 19,835 G1 eTable 2. Unadjusted Odds Ratios (ORs) and 95% Confidence Intervals (CIs) for ADHD in the Third Generation (G2) by Grandmother’s (G0) Joint Exposure to Pre-Pregnancy BMI and GWG, Among the 19,835 Mothers (G1) in the Nurses’ Mothers’ Cohort Study eTable 3. Unadjusted Odds Ratios (ORs) and 95% Confidence Intervals (CIs) for ADHD in the Third Generation (G2) by Grandmother’s (G0) Joint Exposure to Pre-Pregnancy BMI and GWG, Adjusting for Maternal (G1) Pre-Pregnancy BMI, Among the 19,835 Mothers (G1) in the Nurses’ Mothers’ Cohort Study eTable 4. Odds Ratios (ORs) and 95% Confidence Intervals (CIs) for ADHD in the Third Generation (G2) by Grandmother’s (G0) Joint Exposure to Pre-Pregnancy BMI and GWG, Adjusting for Maternal (G1) Pre-Pregnancy BMI, Among the 19,835 Mothers (G1) in the Nurses’ Mothers’ Cohort Study, Stratified by G2 Sex eTable 5. Odds Ratios (ORs) and 95% Confidence Intervals (CIs) for ADHD in the Third Generation (G2) by Grandmother’s (G0) Joint Exposure to Pre-Pregnancy BMI and GWG, Adjusting for Maternal (G1) Pre-Pregnancy BMI and Other Potential Mediators, Among the 19,835 Mothers (G1) in the Nurses’ Mothers’ Cohort Study eTable 6. Data for the Quantitative Bias Analysis of Grandmother Pre-Pregnancy Weight and Grandchild ADHD Status eTable 7. Quantitative Bias Analysis to Assess the Impact of Nondifferential Misclassification of Grandmother (G0) Pre-Pregnancy Underweight in Relation to Grandchild (G2) ADHD in the Nurses’ Mothers’ Cohort Study [file jamanetwopen-e2118824-s001.pdf]

Supplementary Online Content

Yim G, Roberts A, Ascherio A, Wypij D, Kioumourtzoglou MA, Weisskopf MG. Association between periconceptional weight of maternal grandmothers and attention-deficit/hyperactivity disorder in grandchildren. *JAMA Netw Open*. 2021;4(7):e2118824. doi:10.1001/jamanetworkopen.2021.18824

eTable 1. Prevalence of ADHD by Number of Children (Cluster Size) in the Nurses' Health Study II, Among the 19,835 G1
eTable 2. Unadjusted Odds Ratios (OR) and 95% Confidence Intervals (CI) for ADHD in the Third Generation (G2) by Grandmother's (G0) Joint Exposure to Pre-Pregnancy BMI and GWG, Among the 19,835 Mothers (G1) in the Nurses' Mothers' Cohort Study
eTable 3. Unadjusted Odds Ratios (OR) and 95% Confidence Intervals (CI) for ADHD in the Third Generation (G2) by Grandmother's (G0) Joint Exposure to Pre-Pregnancy BMI and GWG, Adjusting for Maternal (G1) Pre-Pregnancy BMI, Among the 19,835 Mothers (G1) in the Nurses' Mothers' Cohort Study
eTable 4. Odds Ratios (OR) and 95% Confidence Intervals (CI) for ADHD in the Third Generation (G2) by Grandmother's (G0) Joint Exposure to Pre-Pregnancy BMI and GWG, Adjusting for Maternal (G1) Pre-Pregnancy BMI, Among the 19,835 Mothers (G1) in the Nurses' Mothers' Cohort Study, Stratified by G2 Sex
eTable 5. Odds Ratios (OR) and 95% Confidence Intervals (CI) For ADHD in the Third Generation (G2) by Grandmother's (G0) Joint Exposure to Pre-Pregnancy BMI and GWG, Adjusting for Maternal (G1) Pre-Pregnancy BMI and Other Potential Mediators, Among the 19,835 Mothers (G1) in the Nurses' Mothers' Cohort Study
eTable 6. Data for the Quantitative Bias Analysis of Grandmother Pre-Pregnancy Weight and Grandchild ADHD Status
eTable 7. Quantitative Bias Analysis to Assess the Impact of Nondifferential Misclassification of Grandmother (G0) Pre-Pregnancy Underweight in Relation to Grandchild (G2) ADHD in the Nurses' Mothers' Cohort Study

This supplementary material has been provided by the authors to give readers additional information about their work.

© 2021 Yim G et al. *JAMA Network Open*.

eTable 1. Prevalence of ADHD by Number of Children (Cluster Size) in the Nurses' Health Study II, Among the 19,835 G1

No. Children (G2)	No. Nurses (G1)	ADHD (%)
1	3,466	315 (9.1)
2	9,819	1,640 (8.4)
3	4,914	1,196 (8.2)
4	1,287	338 (6.6)
5+	349	103 (5.6)

© 2021 Yim G et al. *JAMA Network Open*.

eTable 2. Unadjusted Odds Ratios (OR) and 95% Confidence Intervals (CI) for ADHD in the Third Generation (G2) by Grandmother's (G0) Joint Exposure to Pre-Pregnancy BMI and GWG, Among the 19,835 Mothers (G1) in the Nurses' Mothers' Cohort Study

**Exposure**	**Children (G2)**	**ADHD cases N (%)**	**OR (95% CI)**					
			**Model 1[a]**	***p***	**Model 2[a]**	***p***	**Model 3[a]**	***p***
G0 pre-pregnancy BMI								
< 18.5 kg/m^2	4,712	442 (9.4)	1.25 (1.10, 1.41)	<*.001*	NA	NA	1.25 (1.10, 1.41)	<*.001*
18.5-24.9 kg/m^2	36,915	2,903 (7.9)	1 (Reference)		NA	NA		
≥ 25.0 kg/m^2	3,093	248 (8.0)	0.96 (0.82, 1.13)	0.63	NA	NA	0.96 (0.81, 1.13)	0.62
G0 GWG								
< 20 lbs (9.1 kg)	14,765	1,211 (8.2)	NA	NA	1.04 (0.95, 1.14)	*0.42*	1.04 (0.94, 1.14)	*0.47*
20-29 lbs (9.1-13.2 kg)	18,146	1,436 (7.9)	NA	NA	1 (Reference)		1 (Reference)	
> 29 lbs (13.2 kg)	9,592	780 (8.1)	NA	NA	1.01 (0.90, 1.12)	*0.93*	1.00 (0.89, 1.11)	0.95
Don't remember	2,217	166 (7.5)	NA	NA	1.00 (0.82, 1.23)	*0.97*	1.00 (0.82, 1.23)	0.97

BMI: body mass index; GWG: gestational weight gain

[a] Model 1 presents the total effect of G0 pre-pregnancy BMI in relation to G2 ADHD; Model 2 present the total effect of G0 GWG in relation to G2 ADHD when confounding by G0 pre-pregnancy BMI was not accounted for; Model 3 presents the direct effect of G0 pre-pregnancy BMI in relation to G2 ADHD accounting for G0 GWG and total effect of G0 GWG in relation to G2 ADHD.

© 2021 Yim G et al. *JAMA Network Open*.

eTable 3. Unadjusted Odds Ratios (OR) and 95% Confidence Intervals (CI) for ADHD in the Third Generation (G2) by Grandmother's (G0) Joint Exposure to Pre-Pregnancy BMI and GWG, Adjusting for Maternal (G1) Pre-Pregnancy BMI, Among the 19,835 Mothers (G1) in the Nurses' Mothers' Cohort Study

**Exposure**	**Children (G2)**	**ADHD cases N (%)**	**OR (95% CI)**	***P***
G0 pre-pregnancy BMI				
< 18.5 kg/m^2	4,712	442 (9.4)	1.28 (1.13, 1.45)	*<.001*
18.5-24.9 kg/m^2	36,915	2,903 (7.9)	1 (Reference)	
≥ 25.0 kg/m^2	3,093	248 (8.0)	0.92 (0.78, 1.08)	*0.30*
G0 GWG				
< 20 lbs	14,765	1,211 (8.2)	1.04 (0.94, 1.14)	*0.47*
20-29 lbs	18,146	1,436 (7.9)	1 (Reference)	
> 29 lbs	9,592	780 (8.1)	0.99 (0.89, 1.10)	*0.83*
Don't remember	2,217	166 (7.5)	1.01 (0.83, 1.24)	*0.90*
G1 pre-pregnancy BMI				
< 18.5 kg/m^2	681	49 (7.2)	0.74 (0.54, 1.01)	*0.06*
18.5-24.9 kg/m^2	35,332	2,693 (7.6)	1 (Reference)	
25.0-29.9 kg/m^2	6,483	610 (9.4)	1.21 (1.09, 1.34)	*<.001*
≥ 30 kg/m^2	2,224	241 (10.8)	1.42 (1.20, 1.67)	*<.001*

BMI: body mass index; GWG: gestational weight gain

© 2021 Yim G et al. *JAMA Network Open*.

eTable 4. Odds Ratios (OR) and 95% Confidence Intervals (CI) for ADHD in the Third Generation (G2) by Grandmother's (G0) Joint Exposure to Pre-Pregnancy BMI and GWG, Adjusting for Maternal (G1) Pre-Pregnancy BMI, Among the 19,835 Mothers (G1) in the Nurses' Mothers' Cohort Study, Stratified by G2 Sex*

**Exposure**	**Children (G2)**	**ADHD cases N (%)**	**OR (95% CI)**	***P***
**Among G2 girls (n=20,624)**				
G0 pre-pregnancy BMI				
< 18.5 kg/m^2	2,155	138 (6.4)	1.36 (1.10, 1.69)	0.005
18.5-24.9 kg/m^2	17,065	832 (4.9)	1 (Reference)	
≥ 25.0 kg/m^2	1,404	69 (4.9)	0.91 (0.68, 1.21)	0.51
G0 GWG				
< 20 lbs	6,782	368 (5.4)	1.12 (0.95, 1.33)	0.16
20-29 lbs	8,419	414 (4.9)	1 (Reference)	
> 29 lbs	4,429	219 (4.9)	0.99 (0.82, 1.20)	0.92
Don't remember	994	38 (3.8)	0.93 (0.63, 1.38)	0.72
G1 pre-pregnancy BMI				
< 18.5 kg/m^2	288	15 (5.2)	0.74 (0.43, 1.29)	0.29
18.5-24.9 kg/m^2	16,398	741 (4.5)	1 (Reference)	
25.0-29.9 kg/m^2	2,899	199 (6.9)	1.36 (1.13, 1.64)	<*.001*
≥ 30 kg/m^2	1,039	84 (8.1)	1.66 (1.27, 2.18)	<*.001*
**Among G2 boys (n=22,137)**				
G0 pre-pregnancy BMI				
< 18.5 kg/m^2	2,370	287 (12.1)	1.22 (1.05, 1.43)	0.01
18.5-24.9 kg/m^2	18,237	1,943 (10.7)	1 (Reference)	
≥ 25.0 kg/m^2	1,530	166 (10.9)	0.98 (0.80, 1.19)	0.82
G0 GWG				
< 20 lbs	7,341	784 (10.6)	1.02 (0.91, 1.14)	0.73
20-29 lbs	8,903	958 (10.8)	1 (Reference)	
> 29 lbs	4,753	526 (11.1)	1.02 (0.90, 1.16)	0.76
Don't remember	1,140	128 (11.2)	1.26 (1.00, 1.59)	0.05
G1 pre-pregnancy BMI				
< 18.5 kg/m^2	341	30 (8.8)	0.64 (0.43, 0.97)	0.04
18.5-24.9 kg/m^2	17,396	1,832 (10.5)	1 (Reference)	
25.0-29.9 kg/m^2	3,293	384 (11.7)	1.02 (0.90, 1.17)	0.74
≥ 30 kg/m^2	1,107	150 (13.6)	1.14 (0.93, 1.41)	0.21

BMI: body mass index; GWG: gestational weight gain

*Those without information on G2 sex (933 G0/G1 and 1,959 G2) were not included

Adjusted for grandmother's (G0) race, grandmother's and grandfather's (G0) education, grandfather's (G0) occupation, grandmother's (G0) smoking during pregnancy, grandmother's (G0) alcohol use during pregnancy, grandmother's (G0) lifetime history of depression, and maternal (G1) year of birth

© 2021 Yim G et al. *JAMA Network Open*.

eTable 5. Odds Ratios (OR) and 95% Confidence Intervals (CI) For ADHD in the Third Generation (G2) by Grandmother's (G0) Joint Exposure to Pre-Pregnancy BMI and GWG, Adjusting for Maternal (G1) Pre-Pregnancy BMI and Other Potential Mediators, Among the 19,835 Mothers (G1) in the Nurses' Mothers' Cohort Study

**Exposure**	**Children (G2)**	**ADHD cases N (%)**	**OR (95% CI)**	***P***
G0 pre-pregnancy BMI				
< 18.5 kg/m^2	4,712	442 (9.4)	1.27 (1.12, 1.44)	*<.001*
18.5-24.9 kg/m^2	36,915	2,903 (7.9)	1 (Reference)	
≥ 25.0 kg/m^2	3,093	248 (8.0)	0.95 (0.81, 1.12)	*0.58*
G0 GWG				
< 20 lbs	14,765	1,211 (8.2)	1.05 (0.95, 1.15)	*0.33*
20-29 lbs	18,146	1,436 (7.9)	1 (Reference)	
> 29 lbs	9,592	780 (8.1)	1.00 (0.90, 1.12)	*0.98*
Don't remember	2,217	166 (7.5)	1.13 (0.92, 1.39)	*0.23*
G1 pre-pregnancy BMI				
< 18.5 kg/m^2	681	49 (7.2)	0.69 (0.50, 0.94)	*0.02*
18.5-24.9 kg/m^2	35,332	2,693 (7.6)	1 (Reference)	
25.0-29.9 kg/m^2	6,483	610 (9.4)	1.12 (1.01, 1.25)	*0.04*
≥ 30 kg/m^2	2,224	241 (10.8)	1.21 (1.02, 1.43)	*0.03*

BMI: body mass index; GWG: gestational weight gain

Adjusted for grandmother's (G0) race, grandmother's and grandfather's (G0) education, grandfather's (G0) occupation, grandmother's (G0) smoking during pregnancy, grandmother's (G0) alcohol use during pregnancy, grandmother's (G0) lifetime history of depression, maternal (G1) year of birth, and potential mediators (pregnancy-related complications prior to pregnancy with G2 and G2 birth weight).

© 2021 Yim G et al. *JAMA Network Open*.

eTable 6. Data for the Quantitative Bias Analysis of Grandmother Pre-Pregnancy Weight and Grandchild ADHD Status*

	**Grandmother pre-pregnancy weight**		
**Grandchild ADHD**	**Underweight**	**Normal/Overweight/Obese**	**Total**
Yes	442	3,151	3,593
No	4,270	36,587	40,857

* Uncorrected odds ratio estimate is 1.20 (95% confidence limits, 1.08, 1.33).

© 2021 Yim G et al. *JAMA Network Open*.

eTable 7. Quantitative Bias Analysis to Assess the Impact of Nondifferential Misclassification of Grandmother (G0) Pre-Pregnancy Underweight in Relation to Grandchild (G2) ADHD in the Nurses' Mothers' Cohort Study

**Sensitivity**	**Specificity**	**Misclassification bias corrected odds ratios (95% confidence intervals)**
0.50	0.90	5.35 (2.34, 12.27)
0.50	0.95	1.41 (1.17, 1.69)
0.60	0.90	5.30 (3.23, 12.09)
0.60	0.95	1.39 (1.16, 1.67)
0.70	0.90	5.27 (2.32, 11.97)
0.70	0.95	1.38 (1.16, 1.65)
0.80	0.90	5.24 (2.31, 11.89)
0.80	0.95	1.38 (1.16, 1.64)
0.90	0.90	5.22 (2.31, 11.83)
0.90	0.95	1.37 (1.16, 1.63)

© 2021 Yim G et al. *JAMA Network Open*.
